# Incorporation of patient and public involvement in statistical methodology research: a survey assessing current practices and attitudes of researchers

**DOI:** 10.1186/s40900-023-00507-5

**Published:** 2023-10-27

**Authors:** Lucy Abell, Francesca Maher, Samina Begum, Sarah Booth, Jonathan Broomfield, Sangyu Lee, Ellesha Smith, Rachael Stannard, Lucy Teece, Elpida Vounzoulaki, Hannah Worboys, Laura J. Gray

**Affiliations:** 1 Department of Population Health Sciences, University of Leicester, Leicester, UK; 2 Statistical Methodology PPI Group, University of Leicester, Leicester, UK; 3 Real World Evidence Unit, University of Leicester, Leicester, UK

**Keywords:** Patient and public involvement (PPI), Methodology, Survey

## Abstract

**Background:**

Patient and public involvement (PPI) ensures that research is designed and conducted in a manner that is most beneficial to the individuals whom it will impact. It has an undisputed place in applied research and is required by many funding bodies. However, PPI in statistical methodology research is more challenging and work is needed to identify where and how patients and the public can meaningfully input in this area.

**Methods:**

A descriptive cross-sectional research study was conducted using an online questionnaire, which asked statistical methodologists about themselves and their experience conducting PPI, either to inform a grant application or during a funded statistical methodology project. The survey included both closed-text responses, which were reported using summary statistics, and open-ended questions for which common themes were identified.

**Results:**

119 complete responses were recorded. Individuals who completed the survey displayed an even range of ages, career lengths and positions, with the majority working in academia. 40.3% of participants reported undertaking PPI to inform a grant application and the majority reported that the inclusion of PPI was received positively by the funder. Only 21.0% of participants reported undertaking PPI during a methodological project. 31.0% of individuals thought that PPI was “very” or “extremely” relevant to statistical methodology research, with 45.5% responding “somewhat” and 24.4% answering “not at all” or “not very”. Arguments for including PPI were that it can provide the motivation for research and shape the research question. Negative opinions included that it is too technical for the public to understand, so they cannot have a meaningful impact.

**Conclusions:**

This survey found that the views of statistical methodologists on the inclusion of PPI in their research are varied, with some individuals having particularly strong opinions, both positive and negative. Whilst this is clearly a divisive topic, one commonly identified theme was that many researchers are willing to try and incorporate meaningful PPI into their research but would feel more confident if they had access to resources such as specialised training, guidelines, and case studies.

**Supplementary Information:**

The online version contains supplementary material available at 10.1186/s40900-023-00507-5.

## Abstract (plain language summary)

Patient and public involvement (or PPI) means researchers working in partnership with patients and the public in any part of research. It can include helping decide what the research question is, how to pass on results to the public, and telling researchers what areas are most important to patients and the public. Statistical methods are the tools we use to analyse data. Statistical methodology research involves making sure these tools use our healthcare data in the best way. PPI is essential in health research and is becoming more common in statistical methodology research. But it can be hard to know how to include patients and the public in statistical methodology research. It may seem complex and not directly related to patients. This paper describes the results from a survey we did about the experiences of researchers who have carried out PPI for statistical methodology research. We asked them what they think about it, and how it affects their research. We also asked if they feel confident including PPI in their research, and whether they are given enough help. Researchers had different views about PPI for statistical methodology research. Some people thought PPI was very important in their research, but others weren’t sure. Many people said that they would like more help such as training and guidelines to help them do better PPI in the future.

**Supplementary Information:**

The online version contains supplementary material available at 10.1186/s40900-023-00507-5.

## Background

Patient and public involvement (PPI), defined by the National Institute for Health and Care Research (NIHR) as “research being carried out ‘with’ or ‘by’ members of the public rather than ‘to’, ‘about’ or ‘for them’' [1], allows those who benefit from research to have an active contribution in its design and conduct. PPI is well-embedded in applied health care research in the UK, with research and guidance widely available to inform its conduct across academic, social care and NHS organisations. Much of the guidance available has been initiated by the NIHR, which has made PPI a key requirement of its funded research. This includes non-clinical research, such as statistical methodological advancement. The Medical Research Council (MRC) have recently published a review of public involvement in non-clinical health and biomedical research, within which statistical methodology research falls. The findings highlight the equal importance of public involvement in both clinical and non-clinical health research and provide recommendations for improving practice. Recommendation 8 includes “building consideration of involvement into all funding schemes”, demonstrating the MRC’s commitment to public involvement in their funded research [2].

PPI increases the relevance and moral integrity of research, shifting the focus to be in line with public interest. This is an important aspect of research, since researchers or clinicians may not have first-hand experience of living with a particular disease or attending a particular health service, for example [3]. A literature review aiming to summarise evidence of the impact of PPI in health and social care research found that increased recruitment was observed in all types of research that included public involvement [4]. In the same report, the UK’s Chief Medical Officer at the time stated that PPI “always offers unique, invaluable insights” and can make research “more effective, more credible and often more cost efficient” [4].

Statistical methodology research involves developing, improving, evaluating and comparing statistical methods, including methods for the design, analysis and communication of research studies. Ensuring that researchers are using the right statistical tools to design and analyse studies means that they have the best chance of addressing the correct research questions effectively. Whilst the ultimate aim of this centres on the integrity of research in order to benefit patient care and outcomes, it can be tricky to know how best to involve patients and the public in the development of statistical methodology work. There are unique challenges to incorporate meaningful PPI in statistical methodology research given its technical nature and terminology, and the fact that it is more abstract than applied clinical research. This type of research can be difficult to describe to lay audiences and to get meaningful input into its development which has impact and is not tokenistic.

The purpose of this paper is to summarise the findings of a survey which aimed to assess the attitudes of health sciences researchers towards incorporating PPI within their grant applications or statistical methodology research and assess current practices. From this, we will gain a perspective of the current use of PPI in statistical methodology research, which will help us understand how researchers view PPI. We can then produce targeted resources which aim to improve PPI in statistical methodology research (Additional file 1).

Key objectives of this paper include:

- Identifying stakeholders that are involved in statistical methodology research, specifically healthcare research, including those from relevant university research departments, clinical trials units and industry.
- Establishing current practice and attitudes towards using PPI for statistical methodology research.
- Gathering useful examples/case studies/experience of using PPI in statistical methodology research.

## Methods

This was a descriptive cross-sectional research study using an online questionnaire. The completed GRIPP2 reporting checklist is given in Additional File 1.

### Development of the questionnaire

An online questionnaire was created using Microsoft Forms, containing a range of questions including basic demographics as well as more specific questions relating to the participants’ experience conducting PPI for methodological grant applications and projects. The questionnaire consisted of both closed questions and free-text responses, in order to allow participants to give opinions or recount specific experiences where relevant. A total of 59 questions focused on:

- *Sample characteristics*: 10 questions covering basic demographics as well as those related to career and experience. Participants without any experience with PPI were permitted.
- *PPI to inform a grant application*: 15 possible questions (dependent on responses and their relevant follow-up questions). Specifically, these covered participants’ previous experiences and their opinion on its usefulness in the application.
- *PPI during a funded methodological development project*: similar to questions asked about PPI informing a grant application, with some differences (such as whether the conducted PPI was reflective of the planned PPI). Overall, there were 14 possible questions for participants to answer.
- *Perception of PPI*: participants were asked for their opinions and reasoning regarding relevance, benefits and limitations of PPI in statistical methodology across 9 potential questions. They were also asked about their confidence in conducting PPI and to provide advice for others.
- *General comments*: Four questions asking for any further relevant examples or general comments not previously covered in the survey that participants wanted to include.

The content was informed by an informal PPI methods group set up at the Biostatistics Research Group, University of Leicester and was piloted with a small number of participants prior to being sent electronically to all of the identified participants. This was done to ensure that all questions were clearly worded and to ascertain the approximate length of the questionnaire. The finalised questionnaire was found to take around 10 min to complete and this information was included in the invitation to potential participants in order to encourage participation. The full questionnaire can be accessed in the Additional file 3.

### Participant recruitment

The questionnaire was disseminated initially by contacting individuals at various universities such as biostatistics research groups and clinical trials units (CTUs). Individuals were sent an email invitation including a link to the questionnaire and a participant information sheet. A snowball technique was used by encouraging these individuals to circulate the survey to other members of their group/department, or to provide us with contact details of those that the survey would be relevant to [5]. In addition, mailing lists such as AllStat and social media platforms such as Twitter and LinkedIn were used to advertise the survey. We aimed for a maximum variation sample [5], seeking diversity across numerous areas: workplace (academia, CTUs, industry), region, level of seniority, age and gender. After piloting, the survey was made public on 19th April 2023 and closed on 10th May 2023, allowing 3 weeks for responses to be collected.

### Ethical issues

Ethical approval for this study was received from the University of Leicester before dissemination of the survey. Before completing the survey, participants were asked to confirm that they had read and understood the participant information sheet, which gave details about the project and how their data would be used and hence, provide their informed consent. The participant information sheet can be found in the Additional file 2. All information provided by respondents was stored anonymously and securely and was only accessed by members of the research team. In addition, participants were not obliged to give any personal details unless they wished to be contacted further, ensuring anonymity of responses. All individuals also had the chance to withdraw their responses up until the point when the survey was submitted.

### Analysis

Analysis of the survey results included summary statistics and a descriptive analysis of open-ended questions. Questions requiring a number as an answer were summarised by mean and standard deviation or median and inter-quartile range, depending on the distribution of responses. Multiple choice questions with categorical responses were summarised by number and percentage. Questions where more than one answer could be selected were summarised by the number each potential response was given.

A group of statistical methodology researchers came together to discuss and analyse the 14 free text questions included within the survey. Some of these questions followed on from multiple choice questions and where this was the case, these were included along with the free text response. Responses were printed and read through individually, with common themes and subthemes being identified, along with specific quotes to support these opinions and experiences. This took around 5–6 h, with a total of 11 individuals involved in this analysis. The results of this analysis were presented in tabular form, outlining the themes and sub-themes identified along with quotes to support them.

## Results

Analysis of survey responses was completed based on the sections defined in the questionnaire itself and as such, will be reported in a similar manner. Full tables of closed responses are included in the Additional file 6.

### Sample characteristics

Overall, 122 individuals responded to the survey. One person had completed the survey twice and therefore their second response was removed to prevent duplication of results. Two participants responded “no” to the first question, which asked if they had if they had read the Participant Information Sheet and therefore, they did not complete any more of the survey. Thus, 119 complete responses remained for analysis. The median completion time was 10.25 min (IQR = 5.97, 19.57).

Table 1 shows the demographics of individuals who completed the survey. The age of participants was normally distributed, with 2 participants preferring not to disclose this information. Career length and position were fairly evenly distributed across categories. More respondents were female (58.8%) than male (38.7%), with 3 participants preferring not to say.

**Table 1:** Demographics of participants who responded to the questionnaire

	N = 119
*Age*	
< 25	7 (5.9)
25–34	39 (32.8)
35–44	32 (26.9)
45–54	28 (23.5)
55–65	11 (9.2)
> 65	0 (0.0)
Prefer not to say	2 (1.7)
*Gender*	
Male	46 (38.7)
Female	70 (58.8)
Prefer not to say	3 (2.5)
*Workplace*	
Academia	96 (80.7)
Industry	4 (3.4)
Clinical trials unit	14 (11.8)
Consultancy	1 (0.8)
Other	4 (3.4)
*Career length*	
0–5 years	38 (31.9)
5–10 years	29 (24.4)
10–15 years	20 (16.8)
> 15 years	32 (26.9)
*Position*	
Research Assistant (pre-doc or PhD student)	23 (19.3)
Research Associate (post-doc)	20 (16.8)
Lecturer	25 (21.0)
Senior Research Fellow/Lecturer/Associate Professor	27 (22.7)
Professor	24 (20.2)
*Region*	
North East	5 (4.2)
North West	11 (9.2)
Yorkshire and The Humber	17 (14.3)
East Midlands	33 (27.7)
West Midlands	10 (8.4)
East of England	1 (0.8)
London	11 (9.2)
South East	3 (2.5)
South West	11 (9.2)
Wales	1 (0.8)
Scotland	4 (3.4)
Northern Ireland	0 (0)
Outside UK	10 (8.4)
Other	2 (1.7)
Proportion of day spent on statistical methodology work on average (1–10 scale)	5 (3, 8)
*PPI training undertaken*	
Yes	43 (36.1)
No	76 (63.9)

Values given are median (IQR) for proportion of the day spent working on statistical methodology research and number (%) for all other variables

The majority of respondents worked in academia (80.7%), with several from CTUs (11.7%) and a few from industry (3.4%) or conducting research in the NHS (1.7%). The largest proportion of respondents were based in the East Midlands (27.7%). Otherwise, responses came from all over the UK, including Yorkshire and the Humber (14.3%), West Midlands (8.4%), North West (9.2%), South West (9.2%) and London (9.2%) and some from outside the UK (9.2%).

The main research areas covered by respondents were epidemiology, survival analysis, evidence synthesis, clinical trials and prognostic modelling and the median proportion of day-to-day work spent on statistical methodology was reported as 5/10 (IQR = 3/10, 8/10) on a scale of 1–10. The primary funder of this work was the NIHR, reported by 91 participants (76.5%), with MRC and charities also being common funders. A third (36.1%) of respondents said they had undertaken formal PPI training.

### PPI to inform a grant application

#### Closed-form questions

Of those who completed the survey, 40.3% reported undertaking PPI to inform a grant application for a methodological project. Those who responded “No” to this question (59.6%) justified this either because they had never written/applied for such a grant (27.7%), because PPI was not relevant or required for the project (30.3%) or because there was no clinical application (7.6%). In addition, 18.8% of these responders relayed that they had to justify why PPI had not been conducted in the application.

Common PPI activities conducted by those participants who had undertaken PPI to inform a grant application included: holding a meeting with a group or single person, conducting a survey, receiving feedback on a plain English summary, or receiving guidance on costs for planned PPI activities. Additionally, of these respondents, only 29.2% reported receiving specific funding for their grant development PPI activities and 70.8% were supported by a PPI lead. 62.5% said that their proposal had a clinical application and of these, 90.0% thought that this made it easier to conduct PPI. The median time spent conducting PPI was 5 h (IQR = 4, 10), although some participants stated that it was difficult to quantify this since activities took place over a few months.

Most PPI groups comprised of members of the public and patients, although clinicians and methodologists were also included for some respondents. These individuals were primarily recruited from existing groups and connections, with others from advertising, charities, the NIHR, PPI coordinators or hospital clinics. The most common number of PPI members involved was 5–10 (52.1%), with many using < 5 (37.5%) but fewer using > 10 (10.4%). 58.4% of these participants felt that PPI was very or extremely useful and improved their application and 25.0% felt that it somewhat did.

#### Open-ended questions

Following on from the previous questions outlined above, participants were asked “*Can you describe an example of where it (PPI) had a positive impact?”* and “*Did you receive any feedback from the funder about PPI?”*. Responses are summarised in Table 2. The themes identified in Table 2 show some clear ways in which PPI can aid methodological research projects, including shaping methods and choice of variables and guiding scenarios considered in a simulation study.

**Table 2:** Common themes, subthemes and quotes identified in response to the question “Can you describe an example of where it had a positive impact?” from “Results” and “Discussion” sections of the questionnaire

Type of PPI	Theme	Illustrating quotes
Pre award	Helped frame/refine the research question	"The PPI helped frame the clinical question that accompanied my methodological work, in particular the outcomes used and the language used to describe them" (P47)
	Emphasised importance of work	"It emphasised the clinical importance of the work and highlighted real life concerns of patients" (P35)
		"Contributed for the contextualisation of the problem and to bring to life why it mattered" (P51)
	Identified the importance of data quality/linkage	"They reported about the poor quality of their primary care record and encouraged me to use multiple data sources" (P3)
	Refined outcomes/language used + direction of project	"It helps shape the methods and choices of variables, as well as giving us grounding in the project" (P45)
		"Helped us consider additional potential risk factors/outcomes for our analysis" (P18)
		“Guided the scenarios to be considered during a simulation study and terminology being used to describe the methods.” (P79)
	Improved application in terms of communicating Plain English Summary	"As a result of the meetings I changed my Plain English Summary so that it was easier to understand from a lay perspective" (P5)
	Guided the proposed PPI	"They changed the plans for PPI meetings (if the study was funded) and suggested alternating between in-person and online meetings, having a few static members but cycling through other PPI members to reach a wider and more diverse range of participants. (P23)
	Helped guide costings	"Enables us to properly cost the PPIE involvement in the project which funders really liked" (P98)
During project	Improved communication	“Two members also sit on my stakeholder advisory group so they comment on my decisions at key timepoints and help me to determine how to feed this back to participants.” (P46)
	Helped frame/refine the research question	“Working with care-experienced children was very important and meaningful. Understanding their opinions on what care placements were the most important, along with the key risk factors for education allowed us to think about how we could streamline our analysis” (P68)
	Provided input into modelling	“Suggested an alternative modelling strategy” (P104)
		“Asked for and gained input on design of prediction model tool” (P109)
	Refined materials/tools used	“PPI help to design trial information material” (P52)
		“Improved language used for web-based tool” (P62)
	Provided the bigger picture and clinical perspective	“Forced methodologists to think about the bigger picture” (P33)
		“Added balance to clinical views and different perspectives” (P100)
		“They have always been great at reiterating why the work is important from their perspective which gives a sense of purpose to the projects” (P51)

Participants reported that feedback from funders suggests that PPI creates a good application and that there can never be too much.

"Positive about involving PPI in the design stage of the project" (P5)

"I think they liked that I had a PPI expert helping me with my sessions" (P106)

"Yes, they always want more!!" (P16)

Funders advised applicants to attend PPI training, give more details on how the PPI would work in practice and to increase the number of PPI meetings.

"In the grant reviews the feedback included that I should do some training on working with stakeholders" (P47)

"It also said that the application lacked detail on how the PPI would work in practice" (P47)

"They wanted me to increase the number of PPI meetings I'd planned to conduct during the research from one a year to two a year—it was a condition of the grant being awarded" (P122)

### PPI during a funded methodological development project

This section contained similar questions to the previous but was concerned with PPI carried out during a project rather than to inform a funding application.

#### Closed-form questions

Only 25 (21.0%) participants reported that they had undertaken PPI in this context. Reasons for PPI not being conducted were primarily that it was not required or relevant for the project, or because respondents had not been funded for a methodological project or had not yet reached the part of their project where PPI would be conducted. In addition, some individuals responded that they were unsure how they could involve PPI and faced challenges funding PPI activities.

As before, the most common PPI activities conducted were holding meeting with a group or single person. The median number of hours spent conducting PPI was 6 (IQR = 5, 10), although, as before, some participants stated that it was difficult to quantify this. Of the 25 respondents, 17 (68.0%) reported being supported by a PPI lead. 64% of research projects had a clinical application, and all but one respondent said that this made it easier to conduct PPI. The responses related to the background and recruitment of PPI members reflects the results from the previous section. Additionally, the average number of PPI members used was also similar. 60% of participants felt that PPI was “very” or “extremely” useful and improved their project and 32% felt that it “somewhat” did, with only 2 participants responding “not very” or “not at all” to this question.

Finally, participants were asked whether the PPI conducted was reflective of what they had planned. Only two individuals responded no, due to difficulty in recruiting patients or cutting corners due to a timeline.

#### Open-ended questions

Participants were again asked whether they could provide an example of where PPI had a positive impact. The main themes identified suggest that including PPI ensures a different perspective is provided which can help to refine the aims of the research or the tools used during it. Input into prediction modelling was also specifically mentioned (Table 2).

### Attitudes towards PPI/general comments

Participants were asked “Do you think that PPI is relevant to statistical methodology research?” 31.0% of individuals responded “Very” or “Extremely” to this question with 45.5% responding “Somewhat” and 24.4% answering “Not at all” or “Not very”. A justification was required for these responses, which could generally be grouped into positive, neutral, and negative attitudes (Table 3).

**Table 3:** Common themes, subthemes and quotes identified in response to the question “Do you think that PPI is relevant to statistical methodology work?” from “Conclusions” section of the questionnaire

Attitude group	Theme	Illustrative quote
Positive	Aids in communication of work	“Most of the work that we do is funded by taxpayers, so I think that they should always have a say in our research. It also helps the public know more about what we do (i.e. no more scary statistics!) and improves our own communication skills and how we interact with the public.” (P13)
	Provides motivation for the research	“For statistical methodology research, it is easy to get caught up in the methodology. PPI provides an opportunity for the researcher to get first hand account on how important the topic is to the people that will be affected by the research. They become the motivation for your research.” (P35)
	Shapes the research question and determines prioritisation of work/funding	“Can confirm importance of question and also ensure the approach reflects real-world experiences” (P104)
		“PPI is very important at informing what questions we should ask and where research needs to be directed.” (P42)
		“Many methods projects are not developed in a way that allows the work to be adopted widely and applied for patient and public benefit.” (P103)
	Public confidence/understanding of data	“Any study using patient and public data is inherently linked to the interests of patients and the public. Involvement allows them to have a say in how their data is used.” (P65)
		“If the general public had better understanding of the importance of data sharing and safe guards in place to protect them I hope in the future they are more likely to share there personal data.” (P120)
	Patients/public are often underestimated and can contribute	“Yes, we are using new statistical methods or more advanced methods. It can be tricky but using common language and giving participants an active role in selecting variables, or allowing them to expand on their feelings of certain approaches can offer valuable thinking on how the results are interpreted by the groups who they affect.” (P68)
	Relevance of work to the public	“The methods we use will impact the information they are provided with. Therefore, we need to make sure we are developing methodology which provides answers they are interested in.” (P46)
Neutral	The PPI group should be statisticians	“The target for this work is statisticians, not patients. So eliciting the thoughts of target end users (methodologists) might be helpful.” (P38)
		“The findings are relevant to those with knowledge of the area who are likely to use them, not the layperson.” (P114)
	Dependent on the research	“Sometimes it can be difficult to see where public can meaningfully contribute (or have much interest) if the work is especially theoretical and patient/public implications for quite far down the line. Other methodology work has more obvious implications for public/patients. However, I’d hope public would help identify where they can input meaningfully rather than me dictate that.” (P86)
		“There are some aspects which are very relevant (e.g., choosing appropriate outcome measures and how to present them) and others where it would be difficult for PPI members to contribute meaningfully without specialist training—e.g. comparing different modelling techniques.” (P116)
		“It depends on the project. There is a place for it in methods being developed in a specific clinical area, but I can’t see how the public would be able to comment on a purely methodological statistics project without expert knowledge themselves.” (P79)
		“A lot of statistical methodology work is driven by the need to obtain unbiased answers to questions. This does not necessarily require PPI input
		There are some areas of research where PPI input can help frame the methodological questions addressed, but not all—a blanket requirement for PPI in methodological work risks being a check box exercise.” (P47)
	Not sure on the relevance	“PPI is a confusing world. Should patients be involved? Yes they should. Are the ideas for how to involve them sensible or a good use of money? No they’re not.” (P66)
		“I’ve seen how it can be both helpful and unhelpful, so my thoughts on its relevance are unclear.” (P71)
Negative	The research is too technical for PPI	“Methodological research must be driven by scientific priority. Most methodological research is completely beyond the understanding of a lay person.” (P93)
		“I don’t think it is possible to include patients/public when carrying out high level methodological work without forcing the patient/public to become an ‘expert’ in the area, and defeating the point of PPI.” (P80)
		“It was challenging to convey complex ethical and design concepts to patient partners but the experience was nevertheless useful and rewarding. As much as I would like to, I cannot imagine involving community partners in the more technical statistical projects I have been involved in. These typically require statistical simulation and theoretical development. I guess partners could help refine research questions/objectives but I'm having a difficult time imagining how to engage them in technical aspects.” (P27)
	Patients/public cannot have a meaningful impact	“The statistical methods shouldn’t alter depending on what patients think…” (P119)
		“It doesn’t really inform/change your ideas of what you are planning to do.” (P30)
		“I often struggle to think how inviting patients to a session would help inform the design of the work.” (P96)
	Tick box exercise	“For statistical methodology it feels like a square peg in a round hole…if there is no meaningful way a non-statistician can contribute then this becomes a tick box exercise that can be detrimental to forming the positive relationships required to make PPI effective.” (P40)
	Low priority for patients to know about statistical methodology	“Deriving a standard error isn’t probably on the top 100 things patients care about…” (P32)

Participants were asked what they believed to be the biggest benefit and limitation of PPI in statistical methodology work (Table 4). Generally, it was felt that the benefits of PPI include shaping the research question to ensure that it is of the most relevance to the public, hence reducing research waste and maximising the impact for patients. Participants also reported that it can shape the methods used and aid in the communication of research findings and developments. It was also noted that PPI is important for transparency about the research we do and to ensure that it is reflective of real-world experiences. It was interesting to observe that some of the responses to this question were also of a negative nature, with opinions such as “money wasting”, “ticks a box” and “improving statisticians’ reputations”. This makes it clear that some respondents felt very strongly about this topic.

**Table 4:** Common themes, subthemes and quotes identified in response to the questions “What do you think the biggest benefit/biggest limitation is of PPI in statistical methodology work?” from “Conclusions” section of the questionnaire

Response group	Theme	Illustrative quote
Biggest benefit	Shapes the research question	“To determine whether the question you are trying to answer is actually relevant/interesting to patients/public or not or whether research would be better focused elsewhere.” (P6)
	Shapes the methods used	“Helpful in developing accurate statistical methodology.” (P121)
		“Patient relevant metrics developed.” (P62)
	Reduces research waste	“Making sure that the methodology has an ultimate purpose, rather than wasting funds on somebody’s pet project that won’t have any impact on patient care (no matter how far away that impact is).” (P54)
	Impact on patients	“Helping to ensure the research we conduct is having the best possible impact on patients/public.” (P81)
		“It might be useful to have patient input into whether the new methodology could actually lead to something patients think is useful.” (P69)
	Aids communication of research	“Improved communication of new methods: when you’re working on something you already “get it”, so it’s good for outside input on whether explanations are clear.” (P108)
		“Challenges methodologists to communicate research to broader audiences and consider how their research is important to end users.” (P23)
	Offers new perspectives	“Receiving insights from patients and members of the public: they bring aa whole new perspective and dimension to research, and are likely to contribute observations from their personal experience that academics have not considered.” (P65)
	Ensures research is relevant	“PPI allows us to ask the correct questions that are relevant and important to patients.” (P42)
	Makes research reflective of real-world experience	“In the context of causal inference, regulatory bodies are open to incorporating real-world evidence into their decision making. So PPI would be useful if you thought of your causal analysis as an emulation of a clinical trial.” (P19)
		“Grounding the research in reality, and focusing them on what matters most to patients and/or public.” (P91)
	Transparency	“Transparency about what we do and how we do it.” (P13)
	For funding purposes	“Not clear there is one beyond appeasing funders.” (P40)
	Ticks a box	“May tick a formal box for funders” (P21)
		“Box ticking” (P114)
	Improving statisticians’ reputation	“Communicating to public that statisticians are highly skilled and research methodology is more complex than most people realise, i.e. PR for statisticians rather than gaining useful feedback.” (P38)
		“I see very limited benefit of PPI in this domain” (P93)
	No benefit/not sure	“I have struggled to identify one in the pieces of work I have worked on” (P96)
		“Great way to waste money” (P32)
Biggest limitation	Finding the right participants	“Finding the right level of understanding for a member of the public to be able to comment in a useful way without overloading/confusing them.” (P4)
		“Potential overrepresentation of included groups vs excluded groups.” (P39)
		“Finding people! Very different from clinical trials, where patients and/or patient groups and/or charities are a natural place to find people.” (P76)
	Relevance	“Patient’s opinions don’t affect how well treatments work, which is what statistical methods should be accurately measuring.” (P119)
		“Adopting a ‘one size fits all’ approach to PPI. There are projects where members of the public are unlikely to be able to offer meaningful advice without specialist training—when this is the case the project should not be penalised/judged “harshly by grant bodies/journals, universities etc.” (P116)
		“Hard to see how patients can inform design of simulations comparing different methods. Practical benefits tend to come in later down the line when a researcher wants to develop a model in practice and is deciding the type of model to develop and how to do so.” (P96)
	Using PPI effectively	“It is really tricky to know how to do it meaningfully and not just for the sake of it.” (P81)
		“There is a huge focus on the process not the benefits making it difficult for researchers to focus on the advantages it confers on statistical methodology work.” (P35)
	Communicating to PPI members	“It is challenging/time consuming to appropriately explain to patients/public what you are doing in order to get beneficial feedback” (P6)
		“Difficult to even explain the problem, let alone the solution.” (P2)
	Funding for PPI	“Funding to support grant development.” (P20)
	Finding the time	“Time and understanding.” (P46)
	Methodologists’ attitudes	“Persuading statisticians to listen and learn.” (P99)
	Complexity of methods	“I am not sure that PPI representatives can legitimately comment on complex methodological issues.” (P84)
		“Although I think PPI should be encouraged for some statistical methodology work, I think mandating it could be another barrier when methodology projects are already difficult to get funded. Would often require explanation of complex ideas/methods which may be difficult to convey in a short amount of time (or of little interest to members of the public without a STEM background). (P110)

Limitations to conducting PPI for statistical methodology work included finding the time, the right PPI members and the funding to carry it out. Respondents also noted that it is difficult to conduct PPI meaningfully, and that a reason for this could be that the topics of research may be too technical to explain to a lay audience. These feelings were also shared in the justifications given for the previous question, “Do you think PPI is relevant to statistical methodology work?”, where it was clear that some participants felt PPI could have no meaningful impact in this research area (*“I see very limited benefit of PPI in this domain”* (P32)) and including it in grant applications and methodological projects was simply perceived as a tick box exercise or a *“great way to waste money”* (P32). However, those who responded that PPI was relevant to statistical methodology work echo the benefits discussed above as well as arguing that patients and the public are often underestimated and can provide meaningful contributions.

Those who had a more neutral view towards this topic surmised that the utility of PPI in this area is dependent on the methodological project specifically and that perhaps in some cases the PPI group should be made up of other statisticians who are the target users of the work.

In terms of how confident respondents felt in conducting PPI for methodology research. 17.7% reported feeling “confident” or “extremely confident”, with reasons for this being previous experience in PPI in non-statistics areas, having a dedicated PPI team, attending training courses or simply their own passion and interest in the area. Conversely, 30.3% of respondents felt neutral towards this question and 52.1% felt “not confident” or “extremely not confident”. When asked what would help them to feel more confident, responses included provision of case studies or examples where PPI had an impact, training for statisticians on how to include PPI in methods work specifically and more experience and practice in the area. It was also said that having an existing PPI team or PPI partners would improve confidence. One common response was the request for clear guidelines for PPI in statistical methodology work specifically. In addition, when asked whether they thought there is enough guidance on conducting PPI for methodology research, 108 (90.8%) of individuals responded “No”. The themes found from the responses to this question with specific quotes are reported in Additional file 4: Table S5, which is provided in the additional materials.

Advice provided for those undertaking PPI for statistics methodology research included, undertaking training or involving someone with experience and using existing groups, which reflects the answers to the question about improving confidence in this area (see Additional file 5: Table S6 in the additional material). Others said to involve PPI early, to recruit appropriate members ensuring a diverse group and to listen to the PPI group. Many also just said to give it a go and *“just do it”* (P20).

Finally, participants were asked to provide any further details of PPI work they had undertaken in this area or any further general comments. This provided some useful insight into other relevant projects that are being undertaken in this area:

"We are currently setting up a PPI methodology group… as we have had difficulty with doing this work in the past but think it's really important. This is building on work done… with patients about numerical aspects in trials which was very informative to us" (P22).

“I am putting together a PPI group to steer the interface and contents of a risk communication web tool for patients.” (P1)

"Our methodology project related to trials in rare diseases—it will launch in July 2023 and runs for 5 years—I would be happy to feedback on further experience of PPIE involvement in this once next year when the project is fully underway" (P98).

In addition, the need and want for guidance in this area was expressed:

"This is an area I struggle with a lot, so I'm looking forward to seeing the results." (P108)

"Statisticians need more help and support to conduct statistical methodology work." (P52)

There were also some negative views about the lack of importance PPI has in statistical methodology research:

"Interesting project but overall, I think getting funding for methodological work is hard enough. Adding additional barriers and requirements like PPI does not seem like a good idea to me. It is hard enough to recruit and retain good statisticians, adding additional stuff that post-docs and early career researcher have to learn and master may be counterproductive for our profession." (P76).

## Discussion

We found that statistical methodologists have a wide variety of attitudes, experience and confidence in conducting PPI in statistical methodology research. Participants were generally positive about involvement of PPI and had relevant examples of where it had a positive impact on their research. However, some participants felt that PPI had no place in statistical methodology and should be reserved for applied research. Others thought that methodologists should be the members of the PPI group. The need for more support in conducting PPI was shared by most participants. The development of guidelines, case studies and training were suggested to combat this and to improve researchers’ confidence in this area.

This survey is the first to investigate PPI within statistical methodology research. Previous research has been conducted into PPI in clinical trials methodology and in applied settings [6, 7] Click here to enter text., but no research has investigated PPI in the broader area of statistical methodology as a whole. Although we cannot assess the representativeness of the sample. The characteristics of completers showed a good distribution of ages, career length and career stage. We recruited more females than males, which is not uncommon in online survey research [8]. Participants were recruited mainly from the UK, with wide geographical spread. Although there is over representation of the East Midlands compared to other UK areas.

Overall, attitudes towards PPI were widespread; though there were more positive than negative responses towards its relevance. The majority of participants felt PPI was “somewhat” relevant in statistical methodology research, highlighting the common theme throughout the survey that PPI is useful in certain settings, but less so in others.

Just over half of the participants were not confident in conducting PPI for methodology research. A call for better guidance in the area was echoed by many participants. Participants also requested model examples and case studies for researchers to follow when conducting their own PPI. A limited number of case studies of PPIE activities to inform statistical methodology research are published, as more work is done in this area hopefully this number will increase [7, 9, 10]. Development of guidelines is essential to the improvement of PPI involvement in statistical methodology research. The survey revealed a need for training. Many confident participants in PPI for methodology research attributed their skills to prior training or experience. Others also stated that a dedicated PPI lead ensured that the PPI group provided a more meaningful impact.

We found there were some strong negative views towards PPI. The survey was divisive; participants were split into those who saw the positive impact of PPI in methodology, and those that felt methodology was not an area PPI could contribute meaningfully to. It is therefore essential to improve researchers’ attitudes towards this subject, through areas previously identified (such as guidelines, case studies, examples). Some participants suggested involving methodologists as PPI members, as they are the end users of methodology work. Although important, as researchers are key stakeholders in their fields, this should complement, not replace, PPI involvement.

This first of its kind survey had many strengths. There were a large number of responses which captured a range of opinions. The participants' backgrounds were varied which ensured representation across a broad range of individuals. It was a popular topic and generated discussions on social media platforms, focusing on the usefulness of PPI in statistical methodology. We also piloted the survey and received feedback, before disseminating to a wider audience. This ensured the questions asked would target our aims effectively.

There were some limitations. Since the survey was created and disseminated by the University of Leicester, colleagues were encouraged to complete it, leading to an over representation of views of those from the East Midlands. Participants were perhaps more likely to have stronger views towards PPI, or more experience conducting PPI, as they decided to take part in this survey. This may have resulted in some response bias. Similarly, many respondents said that they had not undertaken formal PPI training and the lack of this may influence their opinions. Other similar surveys have attempted to estimate the level of response bias by obtaining information about the trials that participants have worked on [11]. No bias assessment was possible in this case, but an effort was made to encourage as many people as possible to complete the questionnaire, regardless of their views and experience. Industry reach was limited; however, some survey sections might not apply to them, as they do not have the same funding application process as academia.

As the survey was aimed at all statistical methodologists, a significant proportion of participants did not report conducting any PPI for a grant application or a methodological project, so could not provide any relevant examples or opinions from their experience. However, they still meaningfully contributed to questions about their views of PPI and how to improve its incorporation. The survey asked specific questions and, in many cases, required respondents to convey their feelings through set categories. Conducting interviews with a more qualitative approach could help to reveal deeper themes regarding individuals’ attitudes towards this topic. This approach has been used to elicit views from applied health researchers about PPI [12].

There was no PPI input sought for the creation or dissemination of the survey, as this piece of work was focused on the views of methodology researchers. However, PPI input was obtained when writing the Plain English Summary of this paper, and in offering comments on the survey results. As part of our broader work, we've strived to gather public input on statistical methodology research. Our PPI group were positive about inputting into statistical methodology research and expressed surprise that some statisticians didn’t feel PPI was possible in this area. They felt that, although mathematical ability might help with some aspects of statistical methodology research, everyone can and should be able to be involved in some capacity. Public contributors provide a valuable alternative perspective, helping avoid "blind spots" and enhancing communication in statistical methodology projects. Our public contributors felt that all statistical methodology research should result in patient benefit, even if this only manifests itself later down the line. Therefore, it was deemed that PPI is always important.

Based on this feedback, we thought that it would be interesting to get the public’s reflects on the survey results. We asked a PPI representative for their reflections on the findings of this survey. They felt that, although statistical methodology is a challenging subject area, this does not mean that it cannot be explained with the right interventions and learning support. They stated that there is no need for public contributors to become experts to be able to contribute meaningfully. They were clear about funders increasingly requiring good quality involvement of PPI in research, and the increasingly common inclusion of PPI lay members on funding panels. They also stated that it is not just researchers who would benefit from having guidelines and case studies, but also PPI groups. Providing examples of the variety of ways PPI make a difference, as well as including a PPI lead, would ensure that the PPI group is involved in a meaningful way.

Regarding the attitudes of statistical methodology researchers towards PPI involvement, our PPI representative felt that the more negative views could be due to a lack of training in the involvement of PPI in statistical methodology research. They stated that PPI does not require people to be experts in statistical research, but to have a basic overview of the tools used. An example that our PPI representative gave of good PPI in statistical methodology research, was working on a project where the researchers wanted to communicate risks of frailty to the public. The researcher guided the PPI members through several sessions to produce an infographic, providing greater transparency. Overall, they stated that including PPI in statistical methodology research is not something they would have considered, but can now see the benefits of this, such as increasing confidence in both PPI members and researchers.

The results of the survey have highlighted several areas for further research. There is a clear need for case studies and examples from other researchers demonstrating best practice, as well as training, to help others to conduct their own PPI in methodology research. Respondents also suggested that guidance for conducting PPI in this area would be useful. Such resources may also benefit other areas of research which are removed from direct patient benefit, such as lab-based studies.

## Conclusions

Attitudes towards PPI in statistical methodology research are varied and range from very positive to extremely negative. Many researchers do not feel confident in conducting PPI in this area, and some are yet to be convinced that PPI is even applicable to this research. To encourage more researchers to incorporate PPI and to ensure it has a maximum impact in their research, resources such as case studies, training and the development of guidelines are of great necessity.

### Supplementary Information

**Additional file 1**. GRIPP2 short form checklist. 

**Additional file 2**. Participant information sheet. 

**Additional file 3**. Online questionnaire. 

**Additional file 4. Table S5.** Common themes, subthemes and quotes identified in response to the question “What do you think would help you feel more confident?” from Section 5 of the questionnaire.

**Additional file 5. Table S6:** Common themes, subthemes and quotes identified in response to the question “Do you have any advice for those conducting PPI for statistical methodology research?” from Section 5 of the questionnaire.

**Additional file 6**. Tables of closed responses.

## Data Availability

The datasets used and/or analysed during the current study are available from the corresponding author on reasonable request.

## Abbreviations

PPI(E): Patient and public involvement (and engagement)
NIHR: National institute of health and care research
MRC: Medical research council
CTU: Clinical trials unit
IQR: Inter-quartile range
