# Supplementary material for: Incorporation of patient and public involvement in statistical methodology research: a survey assessing current practices and attitudes of researchers
Source: Res Involv Engagem. 2023 Oct 27;9:100. doi: 10.1186/s40900-023-00507-5 (PMC10612225; doi:10.1186/s40900-023-00507-5)
Supplement: Supplementary file 1 — Additional file 1. GRIPP2 short form checklist. [file 40900_2023_507_MOESM1_ESM.docx]

GRIPP2 short form

| Section and topic | Item | Reported on page No |
| --- | --- | --- |
| 1: Aim | Report the aim of PPI in the study | NA |
| 2: Methods | Provide a clear description of the methods used for PPI in the study | NA |
| 3: Study results | Outcomes—Report the results of PPI in the study, including both positive and negative outcomes | NA |
| 4: Discussion and conclusions | Outcomes—Comment on the extent to which PPI influenced the study overall. Describe positive and negative effects | Pages 19-21 |
| 5: Reflections/critical perspective | Comment critically on the study, reflecting on the things that went well and those that did not, so others can learn from this experience | Pages 18-20 |

PPI=patient and public involvement
