# Supplementary material for: Incorporation of patient and public involvement in statistical methodology research: a survey assessing current practices and attitudes of researchers
Source: Res Involv Engagem. 2023 Oct 27;9:100. doi: 10.1186/s40900-023-00507-5 (PMC10612225; doi:10.1186/s40900-023-00507-5)
Supplement: Supplementary file 2 — Additional file 2. Participant information sheet. [file 40900_2023_507_MOESM2_ESM.docx]

**Participant Information Sheet: Online Survey**

**Title: The use of PPI in statistical methodology research: establishing current practice and attitudes**

Invitation

You have been invited to take part in this research project. Before you decide whether you would like to participate, it is important that you understand why the research is being conducted and what it will involve. Please take your time to read the following information carefully, discuss with others if you wish and ask the researcher if you have questions.

What is the purpose of the research project?

The aim of this research is to establish the current practice of incorporating patient and public involvement (PPI) within statistical methodology research, specifically during the grant application process and throughout the methodology project. This work is part of wider research on this topic being undertaken at the University of Leicester, aimed at encouraging the use of meaningful PPI in methodology research. The ultimate aim of this research is to improve and increase the use and quality of PPI input across statistical methodology research.

Why have I been invited to participate?

You have received this invitation because you have been identified as a statistician who may undertake methodological research as part of your role. You will have received a link to an online questionnaire, sent to you in an email, and by completing the questionnaire you are participating in this research.

Do I have to take part?

It is up to you to decide whether or not you wish to participate in this study. If you decide to complete the online questionnaire you are consenting to take part in this research. The information you share in the online questionnaire will be confidential and anonymous, unless you indicate you would be happy to be contacted by the research team at the end of the questionnaire.

What will happen if I agree to participate?

You will be asked to complete an online questionnaire, which should take 10-15 minutes. By completing the questionnaire you agree to take part in the research. The questionnaire will include a question asking if you are willing to be contacted for further information about your experience with PPI, or to take part in a follow-up interview. If you agree to be contacted then one of our researchers may contact you to discuss this further. You will not be obliged to take part in this research and can decide not to take part when contacted. At all times this project will comply with the General Data Protection Regulations (GDPR, 2018) approved by the EU parliament on 14^th^ April 2016 and passing into UK law with effect from 25^th^ May. If you require more GDPR data protection information, then you can access this via the University’s Information Assurance Services.

Are there any possible disadvantages or risks to taking part?

Participating in the research is not anticipated to cause you any disadvantages or discomfort. The potential physical and/or psychological harm or distress will be the same as any experienced in everyday life. There are some open-ended questions about your experiences of undertaking PPI. If these responses are used in our outputs, we will ensure that no identifiable information about either yourself or the research can be deduced.

What are the potential benefits of taking part?

There is the opportunity to be involved in improving the use and impact of PPI in statistical methodology research. If you agree to be contacted and are invited to interview, you could provide information to be used in educational presentations about PPI research. You may also have the opportunity to provide talks/seminars to present your experiences using PPI or to educate others on how best to incorporate PPI into their research.

Will the information I give stay confidential?

All the information collected about you during this research will be kept strictly confidential. You will not be able to be identified or identifiable in any reports or publications. Any data collected about you in the online questionnaire will be stored online in a form protected by passwords and other relevant security processes and technologies. Data collected may be shared in an anonymised form to allow reuse by the research team and other third parties. These anonymised data will not allow any individuals or their institutions to be identified or identifiable. Identifiable data collected in relation to follow-up interviews will be stored in a password protected Excel spreadsheet on the secure University of Leicester system. This information will be securely disposed of once the participants have been invited to interview. As the data is anonymised, you will not be able to withdraw after completing the questionnaire.

What will happen to the results of the research project?

The findings of this study will be presented at conferences and published in academic journals. You will not be identified in any report or publication.

Who is organising and funding the research?

The project is jointly led by Lucy Abell and Francesca Maher, who are both National Institute of Health and Care Research (NIHR) Pre-Doctoral Research Fellows at the University of Leicester. The project is part of wider research conducted by a group of researchers at the University of Leicester, led by Professor Laura Gray. The project is supported by the National Institute for Health and Care Research (NIHR) Applied Research Collaboration East Midlands (ARC EM) and Leicester NIHR Biomedical Research Centre (BRC).

Who has reviewed the research project?

This project and associated documents have been approved by the University of Leicester Research Ethics Committee.

What happens next?

Please keep this information sheet in a safe place. If you do decide to take part, please click on the link below and complete the questionnaire.

[Link to questionnaire]

Contacts for further information

Lucy Abell, Department of Health Sciences, University of Leicester, UK. Email: [la246@leicester.ac.uk](mailto:la246@leicester.ac.uk)

Francesca Maher, Department of Health Sciences, University of Leicester, UK. Email: [fm234@leicester.ac.uk](mailto:fm234@leicester.ac.uk)

If you have any concerns or queries about the way in which this project has been conducted, then you should contact the Chair of the University Research Ethics Committee on [ethics@le.ac.uk](mailto:ethics@le.ac.uk)
