# Supplementary material for: Incorporation of patient and public involvement in statistical methodology research: a survey assessing current practices and attitudes of researchers
Source: Res Involv Engagem. 2023 Oct 27;9:100. doi: 10.1186/s40900-023-00507-5 (PMC10612225; doi:10.1186/s40900-023-00507-5)
Supplement: Supplementary file 3 — Additional file 3. Online questionnaire. [file 40900_2023_507_MOESM3_ESM.pdf]

The use of PPI in statistical methodology research: assessing current practice & attitudes

* Required

1. I confirm that I have read and understood the participant information sheet for the study and have had the opportunity to ask questions, which have been answered satisfactorily. *

- ◯ Yes
- ◯ No

2. I understand that my participation is voluntary and that I can withdraw at any time up until my survey responses have been confirmed, without giving reason. I understand that once my answers have been submitted, I will not be able to withdraw them, due to the anonymous nature of the data collection. *

- ◯ Yes
- ◯ No

3. I understand that at all times this research project will comply with the *General Data Protection Regulations (GDPR, 2018)* approved by the EU parliament on 14 April 2016 and passing into UK law effective from 25 May 2018 and that if I have any concerns how I contact the University of Leicester to raise these.
   *

   - Yes
   - No

4. I understand that the information I provide will only be accessed by members of the research team and will be stored anonymously and securely. *

   - Yes
   - No

5. I understand that all information will be kept confidential. *

   - Yes
   - No

6. I agree to the uses of the information I provide as detailed in the participant information sheet. *

   - Yes
   - No

7. I agree to take part in the study. *

- Yes
- No

8. Where do you work? *

- Academia
- Industry
- Clinical trials unit
- Consultancy
- Other

9. In which region is your workplace located? *

- North East
- North West
- Yorkshire and The Humber
- East Midlands
- West Midlands
- East of England
- London
- South East
- South West
- Wales
- Scotland
- Northern Ireland
- Outside UK
- Other

10. What is your age? *

- ○ <25
- ○ 25-34
- ○ 35-44
- ○ 45-54
- ○ 55-65
- ○ >65
- ○ Prefer not to say

11. How would you describe your gender? *

- ○ Male
- ○ Female
- ○ Non-binary
- ○ Prefer not to say
- ○ Other

12. How long have you been working in statistical methodology research? *

- ◯ 0-5 years
- ◯ 5-10 years
- ◯ 10-15 years
- ◯ >15 years

13. What level job do you currently work at? (If you don't work in academia please pick the option that best fits your seniority) *

- ◯ Research Assistant (pre-doc or PhD student)
- ◯ Research Associate (post-doc)
- ◯ Research Fellow / Lecturer
- ◯ Senior Research Fellow / Lecturer / Associate Professor
- ◯ Professor

14. How would you describe your main area(s) of research? (Tick all that apply) *

- [ ] Epidemiology
- [ ] Survival Analysis
- [ ] Health Economics
- [ ] Evidence Synthesis
- [ ] Clinical Trials
- [ ] Infectious disease modelling
- [ ] Prognostic modelling
- [ ] Environmental
- [ ] Other

15. How much of your day to day work is spent on statistics methodology? (On a scale of 1-10, with 1 being none and 10 being all of your time) *

16. Who tends to fund the research that you work on? (Tick all that apply) *

- [ ] NIHR
- [ ] MRC
- [ ] UKRI
- [ ] Industry funding
- [ ] Charity funding
- [ ] N/A
- [ ] Other

17. Have you ever undertaken any formal PPI training? *

- ○ Yes
- ○ No

18. Have you ever undertaken any PPI to inform a grant application for a methodological development project? *

- ◯ Yes
- ◯ No
- ◯ Have never applied for such a grant

19. Why not? (Tick all that apply) *

- ☐ Wasn't relevant to the project
- ☐ Wasn't required for the application
- ☐ Project had no clinical application
- ☐ Haven't written a grant application
- ☐ Planned to but couldn't fund
- ☐ Other

20. Did you have to justify in the application why PPI had not been conducted? *

- ◯ Yes
- ◯ No
- ◯ N/A

21. What PPI activities were conducted during this work? (Tick all that apply) *

- ☐ Held a meeting with a group
- ☐ Held a meeting with a single person
- ☐ Received feedback on Plain English Summary
- ☐ Conducted a survey
- ☐ Other

22. How many hours were spent conducting PPI (on average)? *

23. Did you receive any specific funding for this? *

- ◯ Yes
- ◯ No

24. Were you supported by a PPI lead? *

- ◯ Yes
- ◯ No

25. Did your proposal have a clinical application? *

- ◯ Yes
- ◯ No

26. Do you think that made it easier to conduct PPI? *

- ◯ Yes
- ◯ No

27. What was the background of your PPI members? (Tick all that apply) *

- ☐ Patients
- ☐ Public
- ☐ Clinicians
- ☐ Methodologists
- ☐ Other

28. How did you recruit/source them? *

29. What was the average number of PPI members used? *

- ◯ <5
- ◯ 5-10
- ◯ >10

30. Do you feel that the PPI was useful and improved your application? *

- ◯ Not at all
- ◯ Not very
- ◯ Somewhat
- ◯ Very
- ◯ Extremely

31. Can you describe an example of where it had a positive impact?

32. Did you receive any feedback from the funder about the PPI?

33. Have you ever undertaken any PPI during a funded methodological development project? *

- ◯ Yes
- ◯ No
- ◯ Not been funded on a methodological development project

34. Why not? *

- ◯ Wasn't relevant to the project
- ◯ Wasn't required for the project
- ◯ Project had no clinical application
- ◯ Other

35. What PPI activities were conducted during this work? (Tick all that apply) *

- ☐ Held a meeting with a group
- ☐ Held a meeting with a single person
- ☐ Received feedback on Post-Enumeration Survey (PES)
- ☐ Conducted a survey
- ☐ Other

36. How much time was spent conducting PPI (on average)? *

37. Were you supported by a PPI lead? *

- Yes
- No

38. Did your research project have a clinical application? *

- Yes
- No

39. Did this make it easier to conduct PPI? *

- Yes
- No

40. What was the background of your PPI members? (Tick all that apply) *

- [ ] Patients
- [ ] Public
- [ ] Clinicians
- [ ] Methodologists
- [ ] Other

41. How did you recruit/source them? *

42. What was the average number of PPI members used? *

- ◯ <5
- ◯ 5-10
- ◯ >10

43. Was the PPI conducted reflective of the PPI that had been planned? *

- ◯ Yes
- ◯ No

44. Explain what had been planned and why it could not be carried out.

45. Do you feel that the PPI conducted was meaningful and had an impact on your research? *

- Not at all
- Not very
- Somewhat
- Very
- Extremely

46. Can you give an example of where it had a positive impact?

47. Do you think that PPI is relevant to statistical methodology work? *

- Not at all
- Not very
- Somewhat
- Very
- Extremely

48. Please give a justification for your answer. *

49. What do you think the biggest benefit of PPI is in statistical methodology work? *

50. What do you think the biggest limitation is of PPI in statistical methodology work? *

51. How confident do you feel in conducting PPI for methodology research? *

- Extremely confident
- Confident
- Neutral
- Not confident
- Extremely not confident

52. What has helped you to feel this way? *

53. What do you think would help you to feel more confident? *

54. Do you have any advice regarding undertaking PPI for statistics methodology research?

55. Do you think that there is enough guidance on conducting PPI for methodology research? *

- ◯ Yes
- ◯ No

56. If relevant, please provide any further comments or details of PPI work that you have undertaken for a statistical methodological research project.

57. Would you be happy to be contacted regarding your experience of PPI in statistical methodology work in order to discuss it in more detail or be involved in further interviews or workshops? *

- Yes
- No

58. Please provide your name and email.

59. If you would like to be considered for the draw, please leave your email address below:

60. Please include any further general comments you would like to make:

This content is neither created nor endorsed by Microsoft. The data you submit will be sent to the form owner.

Microsoft Forms
