# Supplementary material for: Incorporation of patient and public involvement in statistical methodology research: a survey assessing current practices and attitudes of researchers
Source: Res Involv Engagem. 2023 Oct 27;9:100. doi: 10.1186/s40900-023-00507-5 (PMC10612225; doi:10.1186/s40900-023-00507-5)
Supplement: Supplementary file 4 — Additional file 4. Table S5. Common themes, subthemes and quotes identified in response to the question “What do you think would help you feel more confident?” from Section 5 of the questionnaire. [file 40900_2023_507_MOESM4_ESM.docx]

*Table 5: Common themes, subthemes and quotes identified in response to the question “What do you think would help you feel more confident?” from Section 5 of the questionnaire*

| **Theme** | **Illustrative quotes** |
| --- | --- |
| **Case studies/examples where it has had an impact** | "Some clear examples of where it has been beneficial for statistical methodology work" (P118)    "Seeing some real examples of where PPI has been undertaken and explanation on how it has changed an application (P76) |
| **Training for statisticians/in methods work specifically** | "Training course specifically for statisticians" (P109)    "Formal training around PPI work and embedding PPI in methodological aspects of a trial" (P17) |
| **More experience/practice** | "Time and practice" (P106)    "Experience, shadowing someone else's PP meeting" (P5)    "More experience. I think you have to talk to other people who have done it, then reflect on how it went" (P57) |
| **Having an existing PPI team/partners - both members and research experts working in it** | "Working with a team that does this on a regular basis" (P116)    "Having experienced patient partners and a lead knowledgeable about engagement" (P27) |
| **Clear guidelines (and not just for general PPI but for statistics methodology specifically)** | "Better guidance from funding bodies" (P81)    "I think there needs to be clear guidance on how to conduct PPI for methodological research including examples for how to conduct appropriate PPI when there is no clinical application" (P6)    "Detailed guidance on primarily the aims of PI for methods research... and secondary guidance on how to carry this out, all methodology specific" (P4) |
| **If the public had better awareness of data collection and its importance** | "Public awareness of data collection and its importance" (P88) |
| **Nothing** | "Personality transplant" (P99)    "I don't really care" (P32) |
