# Supplementary material for: Incorporation of patient and public involvement in statistical methodology research: a survey assessing current practices and attitudes of researchers
Source: Res Involv Engagem. 2023 Oct 27;9:100. doi: 10.1186/s40900-023-00507-5 (PMC10612225; doi:10.1186/s40900-023-00507-5)
Supplement: Supplementary file 5 — Additional file 5. Table S6: Common themes, subthemes and quotes identified in response to the question “Do you have any advice for those conducting PPI for statistical methodology research?” from Section 5 of the questionnaire. [file 40900_2023_507_MOESM5_ESM.docx]

*Table 6: Common themes, subthemes and quotes identified in response to the question “Do you have any advice for those conducting PPI for statistical methodology research?” from Section 5 of the questionnaire*

| **Theme** | **Illustrative quote** |
| --- | --- |
| **Give it a go** | "Start small and do not be put off just because something might not have been done before" (P46)    "Just do it. Prepare and circulate any materials in advance. And listen, it's not about you, it's about listening to this group of stakeholders." (P20)    "We will never progress if we don't try! The more we do the more mainstream it will become" (P100) |
| **Undertake training and involve someone with experience** | "Involve someone who is an expert. Get some training. Don't go into it assuming you'll know how to do it properly! And pay accordingly" (P106) |
| **Make sure PPI is necessary** | "It isn't always necessary/useful so think carefully before doing it" (P116) |
| **Establish/use existing groups** | "Have an actual team who specialises in PPI in your institute if possible" (P45) |
| **Listen to the PPI group** | "Contact patient organisations. Start conversations with their representatives" (P59) |
| **Involve PPI early** | "Involve PPIE as early as possible in the grant application process" (P98)    "Plan time and money for PPI activities into grant applications – it takes longer than you think to prepare for PPI meetings" (P122) |
| **Recruit appropriate members** | "The members of the community that will be involved should have basic stats knowledge. The context of the work should be well understood by the participants" (P115) |
| **Ensure a diverse group** | "Very clear communication is essential, different approaches to suit a diverse group, vary the participants, appreciate the participants" (P120) |
