# Supplementary material for: Incorporation of patient and public involvement in statistical methodology research: a survey assessing current practices and attitudes of researchers
Source: Res Involv Engagem. 2023 Oct 27;9:100. doi: 10.1186/s40900-023-00507-5 (PMC10612225; doi:10.1186/s40900-023-00507-5)
Supplement: Supplementary file 6 — Additional file 6. Tables of closed responses. [file 40900_2023_507_MOESM6_ESM.docx]

**Tables of closed responses**

*NB: Number (%) reported for categorical variables, except where more than one response could be given, in which case just the frequency of each response is reported. Median (IQR) is reported for the time spent conducting PPI.*

Section 3

| **Have you ever undertaken any PPI to inform a grant application for a methodological development project?**   - Have never applied for such a grant - Yes - No | 23 (19.3%)  48 (40.3%)  48 (40.3%) |
| --- | --- |
| **Why not?**   - Wasn’t relevant to the project - Wasn’t required for the application - Haven’t written a grant application - Project had no clinical application - Planned to but couldn’t fund - Other | 14  22  10  9  2  9 |
| **Did you have to justify on the application why PPI had not been conducted?**   - Yes - No - NA | 9 (18.8%)  29 (60.4%)  10 (20.8%) |
| **What PPI activities were conducted during this work?**   - Held a meeting with a group - Held a meeting with a single person - Received feedback on Plain English Summary - Conducted a survey - Other | 37  15  36  6  4 |
| **How many hours were spent conducting PPI (on average)** | 5 (IQR = 4, 10) |
| **Did you receive any specific funding for this?**   - Yes - No | 14 (29.2%)  34 (70.8%) |
| **Were you supported by a PPI lead?**   - Yes - No | 34 (70.8%)  14 (29.2%) |
| **Did your proposal have a clinical application?**   - Yes - No | 30 (62.5%)  18 (37.5%) |
| **Do you think this made it easier to conduct PPI?**   - Yes - No | 27 (90.0%)  3 (10.0%) |
| **What was the background of your PPI members?**   - Patients - Public - Clinicians - Methodologists - Other | 38  28  9  3  3 |
| **How did you recruit/source them?**   - NIHR - Charities - Existing groups - Existing connections - Advertising - PPI co-ordinator - Other | 6  5  21  12  7  5  4 |
| **What was the average number of PPI members used?**   - <5 - 5-10 - >10 | 18 (37.5%)  25 (52.1%)  5 (10.4%) |
| **Do you feel that PPI was useful and improved your application?**   - Not at all - Not very - Somewhat - Very - Extremely | 3 (6.3%)  5 (10.4%)  12 (25.0%)  20 (41.7%)  8 (16.7%) |

Section 4

| **Have you ever undertaken any PPI to inform a grant application for a methodological development project?**   - Have not been funded on a methodological project - Yes - No | 35 (29.4%)  25 (21.0%)  59 (49.6%) |
| --- | --- |
| **Why not?**   - Wasn’t relevant to the project - Wasn’t required for the project - Project had no clinical application - Other | 15  23  3  18 |
| **What PPI activities were conducted during this work?**   - Held a meeting with a group - Held a meeting with a single person - Received feedback on Plain Enumeration Survey (PES) - Conducted a survey - Other | 21  9  0  5  2 |
| **How many hours were spent conducting PPI (on average)** | 6 (IQR = 5, 10) |
| **Were you supported by a PPI lead?**   - Yes - No | 17 (68.0%)  8 (32.0%) |
| **Did your proposal have a clinical application?**   - Yes - No | 16 (64.0%)  9 (36.0%) |
| **Do you think this made it easier to conduct PPI?**   - Yes - No | 15 (93.8%)  1 (6.2%) |
| **What was the background of your PPI members?**   - Patients - Public - Clinicians - Methodologists - Other | 20  16  3  1  0 |
| **How did you recruit/source them?**   - Charities - Existing groups - Existing connections - Advertising - PPI co-ordinator - Not involved in recruitment | 2  8  7  4  3  1 |
| **What was the average number of PPI members used?**   - <5 - 5-10 - >10 | 11 (44.0%)  11 (44.0%)  3 (12.0%) |
| **Do you feel that PPI was meaningful and had an impact on your research?**   - Not at all - Not very - Somewhat - Very - Extremely | 1 (4.0%)  1 (4.0%)  8 (32.0%)  9 (36.0%)  6 (24.0%) |
| **Was the PPI conducted reflective of the PPI that had been planned?**   - Yes - No | 23 (92.0%)  2 (8.0%) |

Section 5

| **Do you think PPI is relevant to statistical methodology research?**   - Not at all - Not very - Somewhat - Very - Extremely | 2 (1.7%)  27 (22.7%)  53 (45.5%)  21 (17.6%)  16 (13.4%) |
| --- | --- |
| **How confident do you feel in conducting PPI for methodology research?**   - Extremely confident - Confident - Neutral - Not confident - Extremely not confident | 4 (3.4%)  17 (14.3%)  36 (30.3%)  42 (35.3%)  20 (16.8%) |
| **Do you think there is enough guidance on conducting PPI for methodology research?**   - Yes - No | 11 (9.2%)  108 (90.8%) |
